# The Role of Exosomes in Bronchoalveloar Lavage from Patients with Acute Respiratory Distress Syndrome

**DOI:** 10.3390/jcm8081148

**Published:** 2019-08-01

**Authors:** Tae Hoon Kim, Sang-Bum Hong, Chae-Mann Lim, Younsuck Koh, Eun-young Jang, Jin Won Huh

**Affiliations:** 1 Department of Internal Medicine, Gyeongsang National University School of Medicine and Gyeongsang National University Changwon Hospital, Changwon 51472, Korea; 2 Department of Pulmonary and Critical Care Medicine, Asan Medical Center, University of Ulsan College of Medicine, Seoul 05505, Korea; 3 Biomedical Research Center, Asan Institute for Life Sciences, Seoul 05505, Korea

**Keywords:** acute respiratory distress syndrome, bronchoalveolar lavage, exosome, vascular endothelial growth factor

## Abstract

Background: Acute respiratory distress syndrome (ARDS) is a life-threatening condition caused by pulmonary and extrapulmonary insults. Exosomes are considered a major cell-to-cell communicator and immune modulator. However, their role in ARDS remains unclear. In this study, we investigated whether exosomes could be a potential biomarker of ARDS. Methods: We isolated exosomes from bronchoalveolar lavage (BAL) of patients with ARDS. The correlation between the level of exosomes with clinical data, including etiology, oxygenation, and 28-day mortality was analyzed. Enzyme-linked immune sorbent assays and western blotting were carried out to characterize BAL exosomes. Immune modulating response of exosomes was investigated by in vitro examination. Results: From 158 patients, we isolated mean 1568.9 µg/mL BAL exosomes, which presented a negative correlation with the PaO_2_/FiO_2_ ratio. The level of exosomes did not correlate with 28-day mortality but was elevated in the infectious etiology of ARDS. The exosomes have cargo proteins associated with apoptosis, necroptosis, and autophagy. An in vitro stimulation study revealed that BAL exosomes could induce the production of proinflammatory cytokines and chemokines, but those from patients with ARDS suppressed the production of vascular endothelial growth factor. Conclusions: In ARDS, exosomes are released in alveolar space, and the level is correlated with the etiology of ARDS. BAL exosomes could play an immune-modulating role by controlling the production of cytokines.

## 1. Background

Acute respiratory distress syndrome (ARDS) is a life-threatening condition caused by pulmonary and extrapulmonary insults, increasing alveolar–capillary membrane permeability, and the subsequent interstitial and alveolar edema. Clinically, patients exhibit new or worsening respiratory symptoms; hypoxemia (PaO_2_/FiO_2_ (PF ratio) < 300 mmHg) and bilateral opacity in chest radiography without heart failure [1]. Despite medical advances, hospital mortality was 35–46% in a recent large observational study [2]. Supportive care with mechanical ventilation is still the fundamental approach in ARDS management.

Various cytokines, chemokines, and other mediators that organize the inflammatory cells play an important role in ARDS development [3]. Recently, exosomes are recognized as a major cell-to-cell communicator. Exosomes are generated within intracellular multivesicular bodies and are secreted into the extracellular environment through membrane fusion [4]. Subsequently, they can adhere and fuse to circulating or distant resident cells and transfer a variety of biological molecules [5,6]. Studies have indicated that these exosomal cargos can alter gene expression and regulate immune responses [7,8]. Owing to these characteristics, exosomes are applied as a therapeutic tool and a biological marker for pregnancy and diseases, such as cancer, neurodegenerative disease, cardiovascular disease, and infectious disease [9]. Moreover, studies have suggested that exosomes are involved in the pathogenesis of lung diseases, including chronic obstructive pulmonary disease, idiopathic pulmonary fibrosis, lung cancer, and acute lung injury [10,11,12,13,14,15].

The significance of exosomes in ARDS is not well understood. Therefore, in this study, we aimed to determine whether exosomes could be a potential biomarker of ARDS disease severity or clinical outcome. We also investigated the immune-modulating role of exosomes in ARDS.

## 2. Methods

### 2.1. Subjects and BAL Collection

This study was conducted in a 28-bed medical intensive care unit (ICU) at a tertiary referral hospital in Seoul, Korea between February 2011 and December 2016. Subjects were patients with ARDS who underwent bronchoscopic examination with bronchoalveolar lavage (BAL). ARDS was diagnosed according to the Berlin definition [1]. We obtained clinical data, including the baseline characteristics, respiratory support, arterial blood gas results on the day of bronchoscopy, microbiological results from BAL sample, and 28-day mortality. This prospective study was conducted in accordance with the amended Declaration of Helsinki and approved by the Institutional Review Board (2011-0001) of the Asan Medical Center (Seoul, Korea). Informed consent was obtained from patients or the next of kin.

### 2.2. Exosome Extraction

Exosomes were isolated by differential ultracentrifugation according to the manufacturer’s instructions (Invitrogen, Carlsbad, CA, USA) with some modifications. BAL sample was centrifuged at 1600 rpm to remove cells and debris. The supernatant was centrifuged at 12,000 rpm and passed through a 0.22 µm filter. The supernatant was mixed with the total exosome isolation reagent and vortexed until the solution was homogenous. After incubation for 1 h at 4 °C, the solution was centrifuged at 10,000× *g* for 1 h at 4 °C and the supernatant was obtained. The remnant was centrifuged at 10,000× *g* at 25 °C, and then exosomes pellet was obtained from the bottom of the tube and resuspended in phosphate-buffered saline for further examination.

### 2.3. Western Blot and Enzyme-linked immunosorbent assay

**Figure 1:**
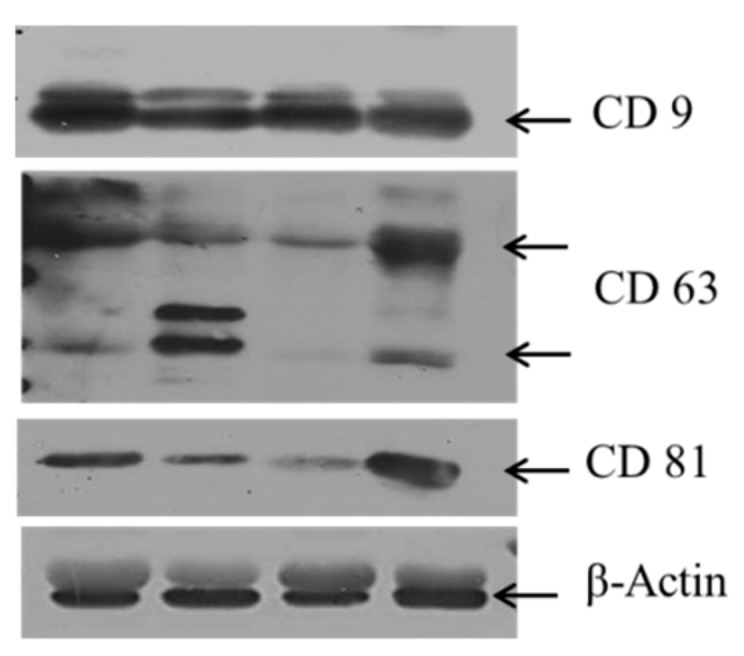
Expression of exosome-associated markers (CD9, CD63, and CD81).

The total protein content in resuspended BAL exosomes sample was measured using a microplate reader (Molecular Devices, San Jose, CA, USA). In the resuspended exosomes pellet, the protein concentrations were determined using the Bradford method. Equal amounts of protein were resolved by electrophoresis on 13.5% sodium dodecyl sulfate–polyacrylamide gel electrophoresis gels and transferred on to polyvinylidenefluoride membranes. After washing with PBST buffer (137 mM sodium chloride, 2.7 mM potassium chloride, and 10 mM phosphate buffer, and 0.1% Tween 20), the blots were blocked with bovine serum albumin, and then incubated with the primary antibodies at dilutions suggested in the manufacturer’s instructions (β-actin, and caspase 12, Santa Cruz Biotechnology; caspase 7, caspase 9, and receptor-interacting protein kinase 3 (RIP3), Cell Signaling Technology, Beverly, MA, USA). The primary antibodies were detected with goat anti-rabbit IgG or goat anti-mouse IgG conjugated to horseradish peroxidase. The intensity of bands was analyzed by enhanced chemiluminescence. In the resuspended BAL exosomes, exosomes-associated markers, such as CD9, CD63, and CD81, were measured using an enzyme-linked immune sorbent assay (ELISA) kit according to the manufacturer’s instructions (ExoELISA Complete Kit; System Biosciences, Palo Alto, CA, USA) and western blot (CD81, Santa Cruz Biotechnology; CD63, Abcam; CD9, Cell Signaling Technology) (Figure 1).

### 2.4. Cell Culture with BAL Exosomes

We obtained BAL exosomes from patients with ARDS and those from controls (patients tolerable in ambient air). Because of the very small number of exosomes from a single patient, BAL exosomes collected from five patients were used to stimulate phorbol-12-myristate-13-acetate (PMA)-differentiated human monocytic THP-1 cells. The cells were treated with BAL exosomes 6 h after PMA treatment. After overnight treatment, cell culture supernatant was obtained and the cytokine/chemokine levels was measured using an ELISA kit according to the manufacturer’s instructions (CC chemokine ligand 2 (CCL2)/ monocyte chemoattractant protein-1 (MCP1), IL-6, vascular endothelial growth factor (VEGF), R&D systems, Minneapolis, MN, USA).

### 2.5. Statistical Analyses

Characteristics of the study subgroups were compared using unpaired *t*-test, Mann–Whitney U-test, as appropriate, for continuous variables, and Fisher’s exact test for categorical variables. Correlations were assessed using Spearman ranked correlation coefficient for non-normally distributed data, and the associated *p* values were calculated. The data are expressed as mean ± standard deviation. For all the tests, results with a *p* value of <0.05 were considered significant. Statistical analyses were performed using software Graph Pad Prism 6 (GraphPad Software Inc., La Jolla, CA, USA) or SPSS 24.0 (IBM Corporation, Armonk, NY, USA).

## 3. Results

### 3.1. PF Ratio and BAL Exosomes

BAL was obtained from 158 patients of mean age 59.5 years; 64.6% of the patients were male (Table 1). Further, 66.5% patients had an infectious etiology and 93.0% of patients were subjected to intubation with a mean PF ratio of 156.1 mmHg (mean FiO_2_ 56.2%). The mean duration of ICU stay was 33.3 days.

The BAL exosomes level was 1568.9 ± 2482.6 µg/mL. The mean concentration of exosome-associated markers in BAL exosomes was as follows: CD9, 0.015; CD81, 0.172; and CD63, 0.137 µg/mL. However, there was no correlation among the exosome-associated markers in BAL exosomes.

**Figure 2:**
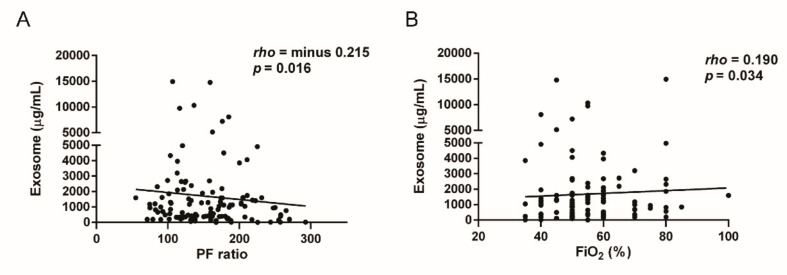
(**A**) Correlation between the level of bronchoalveolar lavage (BAL) exosomes and the PaO_2_/FiO_2_ (PF) ratio. (**B**) Correlation between the level of BAL exosomes and FiO_2_.

To determine the relationship between hypoxia severity and exosomal secretion in ARDS, we compared the PF ratio and BAL exosomes level. The BAL exosomes level significantly correlated with the PF ratio (*rho* = −0.215, *p* = 0.016) and FiO_2_ (*rho* = 0.190, *p* = 0.034) (Figure 2A,B). However, the exosome-associated marker levels did not correlate with the PF ratio (CD9, *p* = 0.791; CD81, *p* = 0.995; CD63, *p* = 0.264).

### 3.2. BAL Clinical Significance and Composition

**Figure 3:**
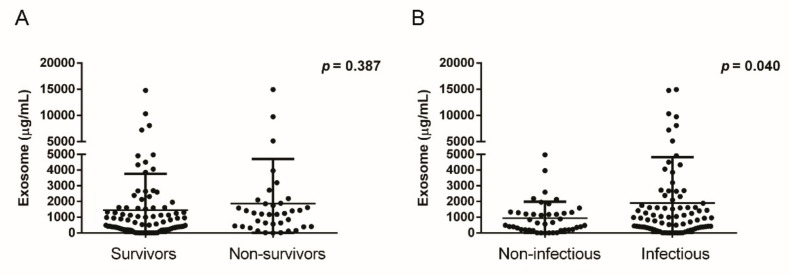
(**A**) BAL exosomes level according to the 28-day mortality of acute respiratory distress syndrome (ARDS). (**B**) BAL exosomes level according to the 28-day mortality or etiology of ARDS.

To investigate the role of BAL exosomes on the survival outcome of patients with ARDS, we divided the patients into two groups depending on 28-day mortality. Forty-nine (31.0%) patients were nonsurvivors; the mean ICU stay was 16.3 days. There was no difference in the PF ratio between the nonsurvivors and survivors (147.2 vs. 160.1 mmHg, *p* = 0.174) on day 1, but the nonsurvivors required a higher concentration of inspired oxygen (60.2 vs. 54.4%, *p* = 0.019). The BAL exosomes level was not different between the nonsurvivors and survivors (1866.8 ± 2844.6 vs. 1443.7 ± 2320.1 µg/mL, *p* = 0.387) (Figure 3A). Additionally, the exosome-associated marker levels did not exhibit significant differences (CD9, 0.014 vs. 0.016 µg/mL, *p* = 0.671; CD81, 0.149 vs. 0.186 µg/mL, *p* = 0.580; CD63, 0.167 vs. 0.098 µg/mL, *p* = 0.401).

The exosome level differed with ARDS etiology. The patients with an infectious etiology presented significantly elevated exosome level, compared with that in patients without an infectious cause (1898.4 vs. 940.7 µg/mL, *p* = 0.040) (Figure 3B). However, there was no difference with age (*p* = 0.065), exosome-associated marker levels (CD9, *p* = 0.800; CD81, *p* = 0.444; CD63, *p* = 0.343), PF ratio (*p* = 0.148), FiO_2_ (*p* = 0.409), or 28-day mortality (*p* = 0.857) between patients with infectious and noninfectious etiologies.

**Figure 4:**
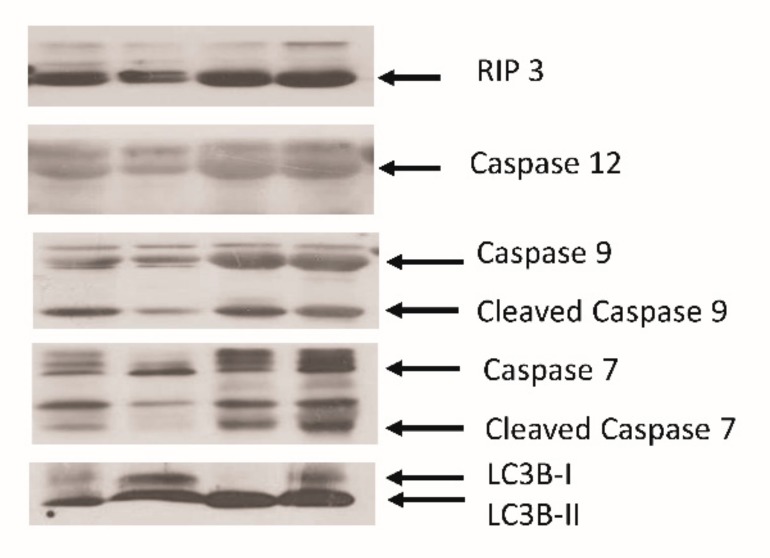
Protein expression in BAL exosomes. Various proteins associated with autophagy, necroptosis, and apoptosis were expressed in nonspecific patterns in BAL exosomes of ARDS patients.

To confirm the exosomes secretion mechanism into the alveolar space in patients with ARDS, we measured the expression of some proteins in BAL exosomes of four patients (Figure 4). Some proteins associated with apoptosis, including caspases 12, 9, and 7, were highly expressed. Additionally, RIP3, a key molecule of necroptosis, was expressed. Finally, microtubule-associated proteins 1A/1B light chain 3B, an important protein in autophagosome formation, was observed. There was no significant difference of expression of these molecules according to the etiology of ARDS.

### 3.3. BAL Exosomes in Immune Response

**Figure 5:**
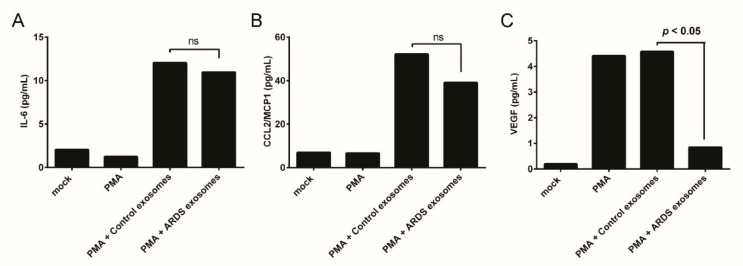
Cytokines and chemokines release by BAL exosome-stimulated cells. (**A**) IL-6, (**B**) CCL2/MCP1, (**C**) VEGF release by BAL exosome-stimulated human monocytic THP-1 cells. PMA: phorbol-12-myristate-13-acetate; ARDS: Acute respiratory distress syndrome.

To investigate the role of BAL exosomes in ARDS on immune cells, in vitro experiments were performed. Exosomes from patients with ARDS had no effect on IL-6 and CCL2/MCP1 secretion compared with those by exosomes from controls (Figure 5A,B). The immune cells stimulated by exosomes from the control produced vascular endothelial growth factor (VEGF), but VEGF production was reduced in cells stimulated with exosomes from patients with ARDS (Figure 5C).

## 4. Discussion

Studies have shown that exosomes are important for cell-to-cell interactions, including proinflammation or immune tolerance functions [6,16]. However, in ARDS, the role of exosomes in BAL is not well-established. Our results demonstrated that a significant number of exosomes are present in the alveolar space during lung injury, and the BAL exosomes level is negatively correlated with the PF ratio. The patients with ARDS due to an infectious etiology had higher BAL exosomes levels than those without an infectious etiology. However, the BAL exosomes level did not differ with 28-day mortality. In an in vitro study, exosomes from patients with ARDS suppressed VEGF production compared with that of exosomes from controls.

In clinical studies, exosomes have been isolated from human BAL in some respiratory disease conditions, such as allergic asthma and sarcoidosis. In our study, the BAL exosomes level was negatively correlated with the PF ratio and was positively correlated with FiO_2_, as evidenced by oxygen-supplying device or ventilator use. Hypoxia could be a major cause of exosomal release in severe hypoxic respiratory failure or inflammatory conditions. A study has shown that intermittent hypoxia can change the components of circulating exosomes in plasma [17]. In a study using breast cancer cells, hypoxia induced exosome release [18]. Contrarily, a treatment-related hyperoxic condition due to external support can induce exosomal release into alveolar and airway spaces. In an animal model, hyperoxia induced the generation and release of extracellular vesicles from lung epithelial cells [19]. Hyperoxia-associated oxidative stress induced extracellular vesicles in mice BAL [20]. We could not conclude whether exosome secretion is due to hypoxia or treatment-related hyperoxia in ARDS. The PF ratio and FiO_2_ showed better correlation with the level of exosomes than PaO_2_, because the range of PaO_2_ was maintained at 55–70 mmHg to maintain the therapeutic target. Our study suggests that the BAL exosomes level is related to oxygen concentration, but further investigation is required to elucidate the exosomal release mechanism.

The exosome level did not correlate with the clinical outcome. However, the nonsurvivors had elevated exosome levels, compared with those of the survivors, but without statistical significance. Particularly, patients with ARDS due to an infectious etiology had elevated BAL exosomes levels. This suggests that the developmental mechanism of exosomes differs with ARDS etiology. Though the exosome level was not evaluated in a large cohort study, there is a possibility that exosomes may be applied as a biomarker for determining antibiotic or steroid treatment in the future.

Exosomes are involved in extracellular vesicle formation, and the diameter of the smallest extracellular vesicle is approximately 30–100 nm [21]. The International Society of Extracellular Vesicles has defined three main subtypes of extracellular vesicles, namely, apoptotic bodies, microvesicles, and exosomes [22]. However, BAL exosomes are different from apoptotic bodies of diameter 500–2000 nm, formed by plasma membrane blebbing during apoptosis, because we isolated BAL exosomes by passing BAL samples through a filter of 0.22 µm pore size. Exosome-specific proteins, such as CD9, CD63, and CD81, were detected. We showed that BAL exosomes from patients with ARDS also expressed some proteins associated with autophagy, necroptosis, and apoptosis. Apoptosis is cell death via signaling molecules. Necroptosis is cell death through trauma by lipid peroxidation of the cell membrane, enzymatic dysfunction, and DNA fragmentation. Our study showed that both the cell death processes are involved in ARDS. Additionally, BAL exosomes from patients with ARDS were associated with autophagy, as reported previously [23]. However, since the expression pattern was nonspecific, it was difficult to explain which pathway plays an important role. Additional studies are needed considering the heterogeneity of etiology.

In the present study, the CD9, CD63, CD81, and CD82 levels in exosomes did not correlate with the BAL exosomes level. This suggests that exosomes from each patient were generated by different mechanisms and cellular source. Potential cell sources of pulmonary vesicles are epithelial cells, alveolar macrophages, endothelial cells, eosinophils, mast cells, platelets, and antigen-presenting cells [16]. Therefore, extracellular vesicle components might be heterogeneously composed depending on the cellular source and environmental stimuli. As CD63 and CD81 are expressed on epithelial cells, bronchial epithelial cells are considered the main source of extracellular vesicles [16,24]. However, in the present study, each patient had different expression patterns of exosomal markers, and we could not determine the main source of BAL exosomes in patients with ARDS.

We evaluated whether BAL exosomes from patients with ARDS stimulate immune cells to produce proinflammatory cytokines and chemokines. In a study on sarcoidosis, a relatively higher amount of *IFN*-g and IL-13 was produced by exosome-stimulated peripheral blood mononuclear cells [25]. BAL exosomes from patients with allergic asthma also promoted the release of LTC4 and IL-8 in human bronchial epithelial cell lines [26]. However, in the present study, VEGF production was attenuated in PMA-differentiated THP-1 cells stimulated by BAL exosomes from patients with ARDS. VEGF has an important role in the pathogenesis of acute lung injury and ARDS, as it can increase permeability and act as a cellular growth factor [27,28,29]. It has been reported that pulmonary-infiltrated Ly6C^hi^ monocytes produce VEGF, which regulates vascular permeability in ventilator-induced lung injury [30]. Additionally, VEGF expression was increased in neutrophils, monocytes, macrophages, and type II pneumocytes in patients with ARDS [31]. This suggests that BAL exosomes might regulate the VEGF production by suppressive cargos. Thus, suppressed VEGF production could affect immune responses and clinical outcomes. However, we could not confirm whether BAL exosomes were absorbed into PMA-differentiated THP-1 cells, and could not analyze exosomal cargos. Currently, anti-VEGF therapy is under investigation for lung cancer, vascular disease, pulmonary hypertension, and chronic inflammatory diseases [32]. The relationship between VEGF and exosomes in ARDS requires further study.

The present study had some limitations. First, this is a single-center study. Second, the low number of subjects might have affected the statistical power of the comparisons. Additionally, this study included patients with various etiologies of ARDS; this heterogeneity could affect the results. Thus, a large-scale study with homogeneity is required to confirm the present study’s results. Third, we could not fully explain the exosome mechanism using in vitro experiments.

Finally, we obtained only a small number of exosomes from each patient’s BAL sample, which made it difficult to reproduce the experiment using identical samples. This is a technical limitation, because mass isolation methodologies and protocols to quantify the absolute number of extracellular vesicles have not been established [5]. Technical improvements are required to accurately understand the role of exosomes and effectively use them. BAL exosomes obtained from ARDS patients and control patients were pooled for in vitro experiments. Exosomes obtained from several patients of the same type of ARDS were mixed to confirm the differential role of ARDS exosomes in in vitro immune stimulation. Nevertheless, to the best of our knowledge, this study is the first to report that exosomes are released to the alveolar space in patients with ARDS and that exosomes could play an important role in ARDS pathogenesis. To know the clinical significance of exosomes, further study of the formation mechanism and components of exosomes is required in various ARDS conditions.

## 5. Conclusions

In conclusion, a significant number of exosomes was released to the alveolar space in patients with ARDS. The BAL exosomes level was positively correlated with hypoxia severity and infectious cause. BAL exosomes from patients with ARDS were composed of various proteins associated with autophagy, necroptosis, and apoptosis. The in vitro study suggested that BAL exosomes may attenuate VEGF secretion. Mechanism studies on BAL exosomes according to the severity and etiology are needed to understand the role of exosomes in ARDS.

## Figures and Tables

**Table 1:** Baseline characteristics of study patients.

Patients Characteristics	*n* = 158
Age (years)	59.5 ± 15.0
Sex (male)	102 (64.6%)
Infectious etiology	105 (66.5%)
Intubation	147 (93.0%)
PaO_2_ (mmHg)	82.0 ± 21.1
FiO_2_ (%)	56.2 ± 14.6
PF ratio (mmHg)	156.1 ± 55.4
ICU stay (days)	33.3 ± 42.1
28 days mortality	49 (31%)
BAL Exosome (μg/mL)	1569.0 ± 2482.6
CD9 (μg/mL)	0.015 ± 0.016
CD81 (μg/mL)	0.172 ± 0.179
CD63 (μg/mL)	0.137 ± 0.156

Abbreviations: PF, PaO_2_/FiO_2_; FiO_2_, fraction of inspired oxygen; ICU, intensive care unit; BAL, bronchoalveolar lavage; Note: Data are presented as numbers (%) or mean ± standard deviation unless otherwise indicated.

## References

[1] Ranieri V.M., Rubenfeld G.D., Thompson B.T., Ferguson N.D., Caldwell E., Fan E., Camporota L., Slutsky A.S. (2012). Acute respiratory distress syndrome: The Berlin Definition. Jama.

[2] Bellani G., Laffey J.G., Pham T., Fan E., Brochard L., Esteban A., Gattinoni L., van Haren F., Larsson A., McAuley D.F. (2016). Epidemiology, Patterns of Care, and Mortality for Patients With Acute Respiratory Distress Syndrome in Intensive Care Units in 50 Countries. Jama.

[3] Reiss L.K., Uhlig U., Uhlig S. (2012). Models and mechanisms of acute lung injury caused by direct insults. Eur. J. Cell. Biol..

[4] Yanez-Mo M., Siljander P.R., Andreu Z., Zavec A.B., Borras F.E., Buzas E.I., Buzas K., Casal E., Cappello F., Carvalho J. (2015). Biological properties of extracellular vesicles and their physiological functions. J. Extracell Vesicles.

[5] Kubo H. (2017). Extracellular Vesicles in Lung Disease. Chest.

[6] Terrasini N., Lionetti V. (2017). Exosomes in Critical Illness. Crit. Care Med..

[7] Robbins P.D., Morelli A.E. (2014). Regulation of immune responses by extracellular vesicles. Nat. Rev. Immunol..

[8] Wang W., Lotze M.T. (2014). Good things come in small packages: Exosomes, immunity and cancer. Cancer Gene Ther..

[9] De Toro J., Herschlik L., Waldner C., Mongini C. (2015). Emerging roles of exosomes in normal and pathological conditions: New insights for diagnosis and therapeutic applications. Front. Immunol..

[10] Takahashi T., Kobayashi S., Fujino N., Suzuki T., Ota C., He M., Yamada M., Suzuki S., Yanai M., Kurosawa S. (2012). Increased circulating endothelial microparticles in COPD patients: A potential biomarker for COPD exacerbation susceptibility. Thorax.

[11] Kadota T., Fujita Y., Yoshioka Y., Araya J., Kuwano K., Ochiya T. (2016). Extracellular Vesicles in Chronic Obstructive Pulmonary Disease. Int. J. Mol. Sci..

[12] Lacedonia D., Carpagnano G.E., Trotta T., Palladino G.P., Panaro M.A., Zoppo L.D., Foschino Barbaro M.P., Porro C. (2016). Microparticles in sputum of COPD patients: A potential biomarker of the disease?. Int. J. Chron. Obstruct. Pulmon. Dis..

[13] Makiguchi T., Yamada M., Yoshioka Y., Sugiura H., Koarai A., Chiba S., Fujino N., Tojo Y., Ota C., Kubo H. (2016). Serum extracellular vesicular miR-21-5p is a predictor of the prognosis in idiopathic pulmonary fibrosis. Respir. Res..

[14] Monsel A., Zhu Y.G., Gudapati V., Lim H., Lee J.W. (2016). Mesenchymal stem cell derived secretome and extracellular vesicles for acute lung injury and other inflammatory lung diseases. Expert Opin. Biol. Ther..

[15] Kadota T., Yoshioka Y., Fujita Y., Kuwano K., Ochiya T. (2017). Extracellular vesicles in lung cancer-From bench to bedside. Semin. Cell Dev. Biol..

[16] Wahlund C.J.E., Eklund A., Grunewald J., Gabrielsson S. (2017). Pulmonary Extracellular Vesicles as Mediators of Local and Systemic Inflammation. Front. Cell Dev. Biol..

[17] Khalyfa A., Zhang C., Khalyfa A.A., Foster G.E., Beaudin A.E., Andrade J., Hanly P.J., Poulin M.J., Gozal D. (2016). Effect on Intermittent Hypoxia on Plasma Exosomal Micro RNA Signature and Endothelial Function in Healthy Adults. Sleep.

[18] King H.W., Michael M.Z., Gleadle J.M. (2012). Hypoxic enhancement of exosome release by breast cancer cells. BMC Cancer.

[19] Moon H.G., Cao Y., Yang J., Lee J.H., Choi H.S., Jin Y. (2015). Lung epithelial cell-derived extracellular vesicles activate macrophage-mediated inflammatory responses via ROCK1 pathway. Cell Death Dis..

[20] Lee H., Zhang D., Zhu Z., Dela Cruz C.S., Jin Y. (2016). Epithelial cell-derived microvesicles activate macrophages and promote inflammation via microvesicle-containing microRNAs. Sci. Rep..

[21] Thery C., Ostrowski M., Segura E. (2009). Membrane vesicles as conveyors of immune responses. Nat. Rev. Immunol..

[22] Crescitelli R., Lässer C., Szabó T.G., Kittel A., Eldh M., Dianzani I., Buzás E.I., Lötvall J. (2013). Distinct RNA profiles in subpopulations of extracellular vesicles: Apoptotic bodies, microvesicles and exosomes. J. Extracell. Vesicles.

[23] Li Z.Y., Wu Y.F., Xu X.C., Zhou J.S., Wang Y., Shen H.H., Chen Z.H. (2017). Autophagy as a Double-Edged Sword in Pulmonary Epithelial Injury: A Review and Perspective. Am. J. Physiol. Lung Cell. Mol. Physiol..

[24] Kulshreshtha A., Ahmad T., Agrawal A., Ghosh B. (2013). Proinflammatory role of epithelial cell-derived exosomes in allergic airway inflammation. J. Allergy Clin. Immunol.

[25] Qazi K.R., Torregrosa Paredes P., Dahlberg B., Grunewald J., Eklund A., Gabrielsson S. (2010). Proinflammatory exosomes in bronchoalveolar lavage fluid of patients with sarcoidosis. Thorax.

[26] Torregrosa Paredes P., Esser J., Admyre C., Nord M., Rahman Q.K., Lukic A., Radmark O., Gronneberg R., Grunewald J., Eklund A. (2012). Bronchoalveolar lavage fluid exosomes contribute to cytokine and leukotriene production in allergic asthma. Allergy.

[27] Senger D.R., Galli S.J., Dvorak A.M., Perruzzi C.A., Harvey V.S., Dvorak H.F. (1983). Tumor cells secrete a vascular permeability factor that promotes accumulation of ascites fluid. Science.

[28] Leung D.W., Cachianes G., Kuang W.J., Goeddel D.V., Ferrara N. (1989). Vascular endothelial growth factor is a secreted angiogenic mitogen. Science.

[29] Kuenen B.C., Levi M., Meijers J.C., Kakkar A.K., van Hinsbergh V.W., Kostense P.J., Pinedo H.M., Hoekman K. (2002). Analysis of coagulation cascade and endothelial cell activation during inhibition of vascular endothelial growth factor/vascular endothelial growth factor receptor pathway in cancer patients. Arterioscler. Thromb. Vasc. Biol..

[30] Shi C.S., Huang T.H., Lin C.K., Li J.M., Chen M.H., Tsai M.L., Chang C.C. (2016). VEGF Production by Ly6C+high Monocytes Contributes to Ventilator-Induced Lung Injury. PLoS ONE.

[31] Maretta M., Toth S., Jonecova Z., Kruzliak P., Kubatka P., Pingorova S., Vesela J. (2014). Immunohistochemical expression of MPO, CD163 and VEGF in inflammatory cells in acute respiratory distress syndrome: A case report. Int. J. Clin. Exp. Pathol..

[32] Medford A.R., Millar A.B. (2006). Vascular endothelial growth factor (VEGF) in acute lung injury (ALI) and acute respiratory distress syndrome (ARDS): Paradox or paradigm?. Thorax.

